# Psychometric properties of the English language version of the C-BiLLT evaluated in typically developing Canadian children

**DOI:** 10.3233/PRM-210101

**Published:** 2023-04-06

**Authors:** Jael N. Bootsma, Fiona Campbell, Dayle McCauley, Sarah Hopmans, Danijela Grahovac, BJ Cunningham, Michelle Phoenix, Olaf Kraus de Camargo, Johanna Geytenbeek, Jan Willem Gorter

**Affiliations:** a School of Rehabilitation Science, McMaster University, Hamilton, ON, Canada; b CanChild Centre for Childhood Disability Research, McMaster University, Hamilton, ON, Canada; c Technology Access Clinic, Developmental Pediatrics and Rehabilitation RJCHC, McMaster Children’s Hospital, Hamilton, ON, Canada; d School of Communication Sciences and Disorders, Western University, Elborn College, London, ON, Canada; e Faculty of Health Sciences, Department of Pediatrics, McMaster University, Hamilton, ON, Canada; f Department of Rehabilitation Medicine, CP Expertise Center, Amsterdam Movement Sciences, Amsterdam UMC, Vrije Universiteit Amsterdam, Amsterdam, The Netherlands; g Department of Rehabilitation, Physical Therapy Science & Sports, UMC Utrecht Brain Center, University Medical Center Utrecht, Utrecht, The Netherlands; h Center of Excellence for Rehabilitation Medicine, UMC Utrecht Brain Center, University Medical Center Utrecht and De Hoogstraat Rehabilitation, Utrecht, The Netherlands

**Keywords:** Cerebral palsy, language comprehension, psychometrics, non-verbal communication, cognition

## Abstract

**PURPOSE:**

This study aimed to 1) investigate the convergent and discriminant validity, internal consistency, and test-retest reliability of the Canadian English version of the Computer-Based instrument for Low motor Language Testing (C-BiLLT-CAN), and 2) explore feasibility of the C-BiLLT assessment for children with cerebral palsy (CP) and complex communication needs in the Canadian health care context.

**METHODS:**

Eighty typically developing children between 1.5 and 8.5 years of age completed the C-BiLLT-CAN, the Peabody Picture Vocabulary Test-IV (PPVT-4), the receptive language sub-test of the New Reynell Developmental Language Scales (NRDLS), and/or the Raven’s 2. Correlations between raw scores were calculated for estimates of convergent and discriminant validity. Internal consistency was calculated for all items and separately for items pertaining to vocabulary and grammar. To calculate the standard error of measurement (SEM) and intraclass correlation coefficient (ICC), 33 participants were re-tested with the C-BiLLT within three weeks. Feasibility was explored with nine participants with CP.

**RESULTS:**

C-BiLLT-CAN’s convergent validity was good to excellent (Spearman’s rho > 0.78) and discriminant validity was higher than hypothesized (Spearman’s rho > 0.8). Internal consistency (Cronbach’s alpha = 0.96), test-retest reliability (ICC > 0.9), and measurement error (SEM < 5%) were excellent. The feasibility study could not be fully completed due to the COVID-19 pandemic. Preliminary data demonstrated some technical and practical barriers for using the C-BiLLT in children with CP in Canada.

**CONCLUSION:**

The C-BiLLT-CAN showed good to excellent psychometric properties in a sample of typically developing children, indicating that it is an adequate test for measuring language comprehension in English-speaking Canadian children. Further research is needed to investigate the feasibility of the C-BiLLT-CAN in children with CP.

## 1 Introduction

Cerebral palsy (CP) causes a disturbance of posture and movement due to a non-progressive brain lesion acquired during early brain development [1]. It affects approximately one in 500 live births, and due to population growth and increased life expectancy, the number of Canadians living with CP is expected to increase in the coming decades [2, 3]. Considerable variation in motor, cognitive, perceptual, and communicative functioning exists in children who share this diagnosis [1, 4–6]. Approximately 16% of children born with CP have extremely limited motor function [7], restricting mobility and speech considerably [8]. CP puts children at risk for intellectual disability and/or specific cognitive impairments, and therefore timely and frequent assessment of functioning across all developmental domains is warranted [9, 10]. With regards to cognitive and language functioning, however, many children with CP are excluded from assessments [11, 12] because of the verbal and motor responses that standard assessment instruments require [13, 14]. While there is growing evidence that the adaptation of response modes (e.g., gaze pointing instead of finger pointing) yields reliable results [12, 15–18], these access methods are rarely incorporated, with serious consequences for research and practice.

In research studies investigating cognitive functioning in children with CP, those with complex communication needs are often either excluded from the sample [19–22] or their abilities are judged based on clinical observation instead of standardized assessment [4]. This paints an incomplete or inaccurate picture of cognitive functioning in this group of children. While correlations exist between severity of motor and cognitive impairments, there is no absolute correspondence [6, 23] and average to gifted cognitive functioning is present across the entire spectrum of motor and speech functioning [12, 23]. The same is true for the development of language comprehension abilities, which may develop typically even if speech is absent [25].

This implies that children’s language comprehension (particularly morphology and syntax) must be accurately assessed so interventions can be tailored to incorporate the individual child’s strengths and address their specific communication challenges [26, 27]. However, for children with CP and complex communication needs, decisions are often made based on observations and clinical judgements [28], which can result in under- or overestimation of language comprehension, causing children to receive services that do not help them reach their full communicative potential.

Originally developed and validated in the Netherlands, the Computer-Based instrument for Low motor Language Testing (C-BiLLT) was designed to overcome the challenges associated with testing children with CP who have low motor and speech function [29]. The C-BiLLT aims to measure the comprehension of spoken words and sentences. The test items are presented verbally, and the answer options are presented visually on a computer screen in a multiple-choice format. The child can select their answer through multiple access methods (i.e., a touch screen, computerized eye-tracking, switch input, or partner-assisted scanning). A higher score on the C-BiLLT indicates better language comprehension skills.

The C-BiLLT’s validity and reliability were assessed in samples of 806 typically developing Dutch children and 87 children with CP and complex communication needs (aged 1 year 6 months [1y6m] -12 years). In the group of children with CP, mean C-BiLLT scores varied widely across the different age groups, but overall the validity hypotheses and reliability parameters were excellent [29].

The current study was part of a larger project examining the cross-cultural validation of the Canadian C-BiLLT (C-BiLLT-CAN), which consisted of the following phases: 1) translation and cultural adaptation of the test, 2) psychometric testing in a sample of typically developing children, and 3) estimating the feasibility of the C-BiLLT-CAN in children with CP and complex communication needs. Phase one was completed prior to the psychometric and feasibility testing according to the guidelines for translating and adapting psychological tests from the International Test Commission [30]. The outcome of phase one was the C-BiLLT-CAN, which was then used for further testing for its psychometric properties. This paper reports results from phases two and three.

The C-BiLLT attempts to measure an individual’s comprehension of spoken language, from single word vocabulary to complex sentences. Because of cognitive and linguistic growth in typically developing children, a significant positive linear trend for age and C-BiLLT-CAN scores was hypothesized. Construct validity of the C-BiLLT-CAN was estimated by testing a priori hypotheses about the correlations between tests with known validity that purport to measure the same construct of spoken language comprehension (convergent validity) and a test that measures non-verbal reasoning (discriminant validity). Hypotheses for convergent and discriminant validity were based on the Dutch validation study [29]. Expected outcomes were: a high correlation (i.e., ≥ 0.8) between the C-BiLLT-CAN and the New Reynell Developmental Language Scales (NRDLS), which measures the same construct, and a slightly lower correlation (i.e., 0.6–0.7) between scores on the C-BiLLT-CAN and the Peabody Picture Vocabulary Test-IV (PPVT-4), a measure of receptive vocabulary. Discriminant validity was assessed using the Raven’s 2, a measure of non-verbal reasoning. A correlation of 0.6 between scores on the C-BiLLT-CAN and the Raven’s 2 was hypothesized.

For a measure to be useful, it must demonstrate sufficient absolute and relative reliability [37]. Relative reliability refers to the degree to which a measure is free from error and remains consistent across administrations, and it is expressed in the intraclass correlation coefficient (ICC) [36]. Absolute reliability, expressed in the standard error of measurement (SEM), refers to the systematic and random error of a measure that is not attributable to true change [36]. SEM is expressed in the same units as the original measurement and represents the confidence interval around a single measurement. This study estimated the relative reliability and absolute measurement error of the C-BiLLT-CAN in typically developing children by retesting participants within three weeks of their first assessment, under the assumption that their level of language comprehension would remain stable over this period. A test-retest reliability (i.e., an ICC of≥0.8) was expected, but an ICC of≥0.6 would be acceptable. A SEM < 10% was considered an acceptably small measurement error.

## 2 Methods

A cross-sectional design was used to estimate validity properties, and a test-retest design was used to estimate the test-retest reliability of the C-BiLLT-CAN in a sample of typically developing children. Feasibility of the C-BiLLT-CAN was explored using a cross-sectional sample of children with CP.

### 2.1 Ethics

The study protocol received ethics approval from the Hamilton Integrated Research Ethics Board (#5152) at McMaster University. Ethical approval to recruit through the Hamilton-Wentworth Catholic District School Board was also received. Parents of all participants provided written informed consent. Participants older than seven years provided written assent.

### 2.2 Participants

Participants for the validation study were recruited via flyers, social media, day care centres, and schools in Hamilton, ON, Canada. Between January 2019 and March 2020, all assessments took place in-person at McMaster University. Due to the COVID-19 pandemic, the protocol was adapted to allow for virtual assessments via Zoom, which took place between July and November 2020. By adding the option to conduct virtual assessments, recruitment could be broadened to allow for assessments to be done across Canada.

Children were eligible for this study if they (1) were between 1.5 and 8.5 years of age; (2) spoke English; and (3) had at least one parent/caregiver who spoke English as their first language. Participants were excluded from the study if they had (1) a history of speech and/or language delay or disorder; (2) a history of auditory and/or visual impairment; (3) a developmental delay or disorder; and/or (4) a neurological or chronic disorder. Data from one participant in the lowest age group were removed because the participant obtained a score of zero, due to distractibility. The sample of typically developing children was thus comprised of 80 children (Table 1). The majority of participants were assessed in-person (n = 50, 62.5%).

**Table 1:** Demographic data of the sample

		TD (*N* = 80)	CP (*N* = 9)
Sex
	Female	45	5
	Male	35	4
GMFCS level
	III	n/a	1
	IV	n/a	2
	V	n/a	6
Language exposure
	English only	43	4
	English &French	14	1
	English &other	11	3
	≥3 languages	12	1
Annual household income
	≤49,999	6	2
	50,000–99,999	13	2
	100,000–149,999	23	1
	≥150,000	34	2
	Don’t know	0	2
	Prefer not to answer	4	0

*Notes:* GMFCS, Gross Motor Functioning Classification System; TD, typically developing; CP; cerebral palsy.

Participants for the feasibility study were recruited through clinics at Hamilton Health Sciences. Children were eligible to participate if they were between 1y6 m - 16 years of age, had a diagnosis of CP, had no functional speech, and were classified as level III-V on the Gross Motor Functioning Classification System (GMFCS). At the start of the COVID-19 pandemic, data collection was abruptly discontinued because of the need for in-person assessments with these participants. The final sample therefore included nine children with CP (Table 1).

### 2.3 Measures

Use of the different measures depended on the eligible ages for the additional tests and the type of study visit (i.e., in person or virtual). Therefore, sample sizes for the different analyses varied (Table 2).

**Table 2:** Sample characteristics for the different analyses

Analysis	# of participants	Age min-max
	(female)
Internal consistency	80 (45)	1y 6 m –8y 6 m
Test-retest reliability and SEM	33 (19)	1y 9 m –8y 6m
Convergent validity
NRDLS	41 (24)	2y 1 m –7y 5m
PPVT-4	70 (44)	2y 6 m –8y 6 m
Discriminant validity
Raven’s 2	33 (20)	4y 1 m –8y 6 m
Feasibility	9 (5)	32 m –10y 6m

*Notes:* NRDLS, New Reynells Developmental Language Test; PPVT, Peabody Picture Vocabulary Test –4th edition; SEM, standard error of measurement.

#### 2.3.1 C-BiLLT

The C-BiLLT is an 88-item test that assesses a child’s understanding of spoken language, with a higher score indicating better language comprehension [29]. The C-BiLLT consists of web-based software that can be combined with several different access methods. Access methods using direct selection include a touch screen and eye gaze computer control. Indirect selection methods include input switches and partner-assisted scanning. Administration of the C-BiLLT follows three parts. The first part is a pre-test in which the child is first asked to identify concrete familiar objects held up by the assessor in sets of two, and then identify the same objects presented as photographs. The next two parts are the computer-based components of the assessment, which test vocabulary, morphology, and syntax by asking the participant to select from a choice of 2–4 the picture that matches the item orally presented by the examiner (e.g., “Which one is the . . . ?”).

Measurement properties of the Dutch and the Norwegian adaptations (C-BiLLT-NOR) show good construct validity, excellent internal consistency, and optimal reliability in samples of typically developing children and in Dutch children with CP [17, 29]. For the original C-BiLLT, exploratory factor analysis (EFA) of a former 75-item version resulted in one factor, labelled comprehension of spoken language, explaining 76% of the variance. For the C-BiLLT-NOR, EFA resulted in a two-factor solution (receptive vocabulary and receptive grammar) that explained 68.6% and 16.6% of the variance in the data, respectively.

The original C-BiLLT was translated into English and adapted for use in Canada. To ensure that the instrument would measure the same phenomenon in the target language and culture (i.e., was equivalent to the original measure), a careful and thorough cross-cultural adaptation process was completed [31]. Guidelines provided by the International Test Commission [32] were followed during the translation process.

#### 2.3.2 PPVT-4

The PPVT-4 [33] is a widely used, untimed instrument that measures single word receptive vocabulary in individuals aged 2.5 years and older. The examiner orally presents a word, and the participant is asked to identify the corresponding picture from a choice of four pictures. For the online study visits, the digital version of the PPVT-4 was used with participants of eligible age.

#### 2.3.3 NRDLS

The NRDLS [34] is a clinical instrument designed to measure comprehension and production of spoken language in children aged 3 years - 7y6 m. It is comprised of 10 subtests, of which eight also measure comprehension. In the present study, these eight subtests were administered to participants of eligible age. The test uses both toys and a picture booklet to elicit responses. There is no digital version of the NRDLS, so this test was not administered during online study visits.

#### 2.3.4 Raven’s 2

The Raven’s 2 [35] assesses non-verbal reasoning in individuals aged 4–90 years. It consists of visual geometric designs of increasing difficulty, each with a missing piece. Participants over the age of four years were asked to identify the missing piece from a choice of five options. For the online study visits, the digital version of the Raven’s 2 was used. However, only scores obtained during in-person study visits could be included, as the digital version did not yield raw scores.

### 2.4 Procedure

To avoid a learning effect, test sessions started with the C-BiLLT-CAN for all participants. Depending on their age, participants were administered one to three additional measures.

Following the C-BiLLT-CAN, measures were presented in two different test orders, to which participants were randomly assigned. Parents could be present during the study visit. Participants received a junior scientist certificate and a $20 gift card for their participation. Thirty-three participants were retested with the C-BiLLT-CAN within three weeks of the original test date. Participants with CP were assessed with the C-BiLLT-CAN, and also with the PPVT-4 if time permitted and a reliable response was achievable (e.g., by pointing).

### 2.5 Examiners

Examiners for the assessments of typically developing children were speech-language pathology graduate students from McMaster University (*n* = 10) who were trained in the administration of standardized language tests and received a minimum of two hours of additional training on the specific tests included in this study. The assessments of children with CP were done by an experienced speech-language pathologist familiar with Augmentative and Alternative Communication (FC).

### 2.6 Assessment of measurement properties

Validity is defined as “the degree to which an instrument truly measures the construct(s) it purports to measure” [36]. This study reports on the convergent and discriminant validity of the C-BiLLT-CAN and its absolute and relative reliability when used with typically developing Canadian children.

### 2.7 Statistical analyses

Data were assessed for normality by visual inspection of QQ-plots and tests of skewness and kurtosis. In many age groups, there was moderate skewness and kurtosis; in some age groups (e.g., 5y6 m - 5y11 m and 6y6 m - 6y11 m), they were high.

Because of these distributions and the small sample sizes per age group, non-parametric measures were deemed more appropriate. Therefore, Spearman’s rho was used to assess validity, and the Jonckheere-Terpstra test was performed to assess the hypothesized trend between increasing age and C-BiLLT-CAN scores. For validity hypothesis testing, one-tailed tests set to a 0.1 significance level were performed, and 99% lower bound estimates were based on Bonett and Wright (2000). Because of the two-factor solution that was found in the C-BiLLT-NOR, Cronbach’s alpha was calculated for items pertaining to grammar and vocabulary separately. The ICC was calculated using a two-way random effects model with absolute agreement. Absolute reliability was calculated as SD-Ö(1-ICC). All statistical analyses were performed using SPSS version 26.

## 3 Results

Participant sample sizes and sex distribution for the different analyses are presented in Table 2. Mann-Whitney U tests showed that the distribution of C-BiLLT-CAN scores did not significantly differ between males (Mdn = 72) and females (Mdn = 72), *U* = 730, *z* = –0.558, *p* = 0.577, nor was there a difference between scores for children who participated in virtual (Mdn = 73) versus in-person study visits (Mdn = 71.5), *U* = 776.5, *z* = 0.264, *p* = 0.792. The sample performed substantially above the population mean on the PPVT-4, mean (SD) Z-score = 1 (0.83). On the NRDLS and the Raven’s 2, the sample obtained a mean (SD) Z-score of 0.53 (0.89) and 0.13 (1.38), respectively. Table 3 shows the distribution of C-BiLLT-CAN scores per age group. A Jonckheere-Terpstra test showed a statistically significant increasing monotonic trend in C-BiLLT-CAN scores, *p* < 0.0005, Kendall’s τb = 0.751.

**Table 3:** Median, minimum (min), maximum (max), and mean (M) with 95% confidence intervals (CI), standard deviation (SD), skewness, and kurtosis per age group of raw scores on the Computer Based instrument for Low-motor Language Testing-Canada (C-BiLLT-CAN)

Age group	*n*	Median (min-max)	M (SD)	95% CI for mean	Skewness	Kurtosis
1;5–1;11	4	41.5 (37–43)	40.8 (2.9)	36.3 –45.3	–0.86	–1.29
2;0 –2;5	6	44 (26–70)	46.2 (13.5)	31.0 –61.4	0.53	1.56
2;6 –2;11	5	60 (52–65)	58.6 (5.1)	52.2 –65.0	–0.17	–1.17
3;0 –3;5	8	62 (47–67)	60.3 (7.2)	54.3 –66.3	–0.92	0.12
3;6 –3;11	5	68 (64–73)	67.8 (3.6)	63.4 –72.2	0.60	–0.23
4;0 –4;5	5	70 (64–74)	69.4 (3.7)	64.8 –74.0	–0.48	0.59
4;6 –4;11	7	72 (64–78)	70.6 (4.4)	66.5 –74.7	0.27	0.67
5;0 –5;5	5	73 (71–82)	74.8 (4.4)	69.3 –80.3	1.39	1.58
5;6 –5;11	5	75 (67–79)	74.6 (4.7)	68.7 –80.5	–1.25	1.66
6;0 –6;5	8	76.5 (71–81)	75.1 (3.4)	73.3 –79.0	–0.29	–0.72
6;6 –6;11	6	81 (71–83)	79.7 (4.6)	74.9 –84.4	–1.80	3.45
7;0 –7;5	5	82 (78–83)	81.2 (2.3)	78.5 –83.9	–0.91	–0.74
7;6 –7;11	5	76 (74–80)	76.8 (3.0)	73.0 –80.6	0.32	–3.08
8;0 –8;5	6	82.5 (80–86)	82.7 (2.3)	80.4 –84.9	0.46	–0.30

*Notes:* CI, confidence interval; ^a^Years;months.

### 3.1 Convergent and discriminant validity

To estimate convergent and discriminant validity, one-tailed Spearman’s rank-order correlations were run to assess the relationship between scores on the C-BiLLT-CAN, NRDLS, PPVT-4, and Raven’s 2 (Table 4).

**Table 4:** Spearman’s rho correlations (99% lower confidence bound) of raw scores on the C-BiLLT-CAN, NRDLS, PPVT-4, and Raven’s 2

	NRDLS	PPVT-4	Raven’s 2
C-BiLLT-CAN	0.780* (0.451)	0.845* (0.630)	0.871* (0.604)
NRDLS		0.627* (0.292)	0.681* (0.257)
PPVT-4			0.747* (0.359)

*Notes:* C-BiLLT-CAN, Computer Based instrument for Low-motor Language Testing-Canada; NRDLS, New Reynells Developmental Language Test; PPVT, Peabody Picture Vocabulary Test –4th edition. *p < 0.001

### 3.2 Internal consistency

Cronbach’s alpha of the C-BiLLT-CAN was calculated for all 88 items (0.960) and separately for the 34 vocabulary items (0.875) and the items pertaining to morphology and syntax (0.948).

### 3.3 Test-retest reliability and SEM

Thirty-three participants were retested with the C-BiLLT-CAN within approximately three weeks of their first test (range 5–26 days, mean 14 days). Mean score at baseline was 68.9 (SD = 13), and mean score at the retest was 75.8 (SD = 10). ICC was 0.96 (95% CI 0.88 –0.98), which indicates excellent reliability [39], and a SEM of 2.3 points, which is < 5% of the possible total score of 88.

### 3.4 Feasibility

The C-BiLLT-CAN could be fully completed for four out of nine participants with CP (three within a single session) and partially completed for the other five. For two, a second session was required but could not be scheduled due to COVID-19 restrictions, and for one participant, a reliable access method for the computer-based parts of the assessment could not be determined. For the two remaining participants with whom the assessment could not be completed in one or two sessions, a second or third session was deemed too burdensome and was therefore not scheduled.

The nine participants used 10 different access methods including touch screen (*n* = 3), eye tracking technology (*n* = 1), switch buttons (*n* = 2), finger, eye and/or body part pointing with target selection confirmed by examiner (*n* = 3), and head mouse with target selection confirmed by examiner (*n* = 1). Six participants used one access method. One switched from touch screen to pointing with target selection confirmed by the examiner after fatiguing, and one participant started with eye gaze, then used the switch button, and ended with pointing with target selection confirmed by the examiner.

## 4 Discussion

This study estimated construct validity, internal consistency, test-retest reliability, and

measurement error of the C-BiLLT-CAN in a sample of typically developing Canadian children. Feasibility of the instrument in the Canadian context was explored in a small sample of children with CP and complex communication needs.

The hypotheses about construct validity were partially confirmed. Convergent validity was excellent between the C-BiLLT-CAN and the NRDLS, indicating that the test can be regarded as a valid measure of language comprehension. The higher than expected correlations between the C-BiLLT-CAN and PPVT-4 (vocabulary) may be explained by the age of the sample. In young children, cognitive abilities are less well differentiated and do not develop in isolation [40, 41]. Comprehension of vocabulary and sentences can best be characterized as a single construct in young (pre-kindergarten to grade 3) typically developing children [42, 43]. This could also explain the high correlation between scores on the Raven’s 2 (non-verbal reasoning) and the C-BiLLT-CAN. Furthermore, because of the small sample size (i.e., only scores of participants who completed the paper version of the Raven’s 2 could be used, *n* = 31), this analysis may have been underpowered [44].

The sufficiently high test-retest reliability indicated that the C-BiLLT-CAN results were consistent for participants whose abilities had not changed over time. The C-BiLLT-CAN’s good internal validity indicated that the different test items measured the same construct in the sample.

The aim of the feasibility study was not achieved because of the need to abruptly terminate this project due to COVID-19 restrictions in 2020. Therefore, the feasibility of the C-BiLLT-CAN in children with CP and complex communication needs is yet to be fully investigated.

However, the data that were collected do suggest the need to carefully consider the local context when ‘moving’ an assessment instrument from one language, culture, and country to another. While health care services may be comparable between Canada and the Netherlands, the geographical (and thus travel times to clinics), organizational, and clinical differences are large. As an example of a geographical consideration, assessments in this trial were done at one clinic, which meant many child participants had to travel for hours. This may have caused fatigue to the extent that the assessment had to be done in two sessions or could not be completed at all. In the Netherlands, many of the children with CP were assessed in their own schools or day care centres, which was feasible for administrators because of the short distances. Additionally, the multiple access methods that were used by the participants in the current feasibility study may reinforce the need for the flexibility and accessibility of the C-BiLLT. For example, a cultural clinical practice difference was demonstrated that needs to be addressed: the head mouse, which is a popular access method in Canada, is not yet part of the C-BiLLT’s access repertoire because it is prescribed much less frequently in the Netherlands. To promote the uptake and use in clinical practice of the C-BiLLT-CAN, the team has proposed an implementation study to explore the factors that will support or hinder effective use of the test in Canadian clinical practice.

There are several strengths of the current study. A priori levels of acceptable and desired construct validity and test-retest reliability were demonstrated. In response to the COVID-19 pandemic, the project was quickly adapted to virtual data collection, which allowed for completion of most of the project as intended.

This study also had some major limitations. The validity of an assessment tool should be estimated if the tool is applied in a new situation or for another purpose [36]. Here, the new situation was the new language and cultural adaptation of the C-BiLLT-CAN. To test if this new version measured what it purported to measure, the C-BiLLT-CAN was validated on a sample of 80 typically developing English speaking Canadian children. In the adaptation process, care was taken to select items and images that were present in the world of Canadian children with CP and complex communication needs, to ensure that the items would be familiar to them. It is important to recognize that validity of the C-BiLLT-CAN in the population of children with CP and complex communication needs has not yet been assessed directly. It should be noted, however, that this will be evaluated by this team as part of a recently funded study and that the validity parameters for the original C-BiLLT with a sample of 87 children with CP and complex communication needs are encouraging. The said four-year research project commences in 2022 and aims to (1) understand Canadian clinicians’ and families’ perceived barriers and facilitators to using the C-BiLLT-CAN, and how they would use results to inform service delivery and education plans; (2) modify and test the C-BiLLT-CAN’s accessibility with Canadian children to ensure that all children have access to a reliable assessment of their language comprehension; and (3) develop and pilot training materials and methods to support implementation in Canada.

The same limitation was present for the assessment of the C-BiLLT-CAN’s reliability. Reliability of an instrument depends highly on the distribution of the characteristic (i.e., language comprehension) in the population (i.e., children with CP). It is possible that language comprehension abilities are distributed differently in a population of typically developing children, and that the reported reliability of the C-BiLLT-CAN in this study may therefore differ if tested in a sample of children with CP. Future research will also assess reliability of the C-BiLLT-CAN in a sample that reflects the test’s target population.

Despite efforts to recruit a balanced sample, the typically developing participant group had above average cognitive functioning, potentially limiting the generalizability of the findings. Furthermore, it is unfortunate that the assessments for children with CP could not be adapted in response to the pandemic. The necessary health safety precautions and in person guidance during the assessments with these participants could no longer be provided in accordance with COVID-19 regulations. As part of the proposed implementation study, local pediatric therapists will be trained to collect data by administering the test with children on their caseloads. This could circumvent children’s exposure to unknown clinicians and extra study visits. Additionally, feasibility of the C-BiLLT-CAN was evaluated in children with CP older than three years. Because of the cognitive challenges associated with indirect access (e.g., attention, timing), it is important that future studies look at younger children with CP as well.

This study’s findings add to the accumulating evidence and need for translated and adapted versions of the C-BiLLT instrument, and are highly anticipated by scientific, clinical, and family end users (45,46, personal communication).

The assessment of measurement invariance of the different versions of the C-BiLLT by confirmatory factor analysis or item response theory techniques is a logical next step for psychometric testing. Knowing whether the different versions of the C-BiLLT function similarly would allow for interesting international comparisons. Results from the feasibility study also call for explicit consideration of the context in which testing is meant to happen, so while there is scientific and clinical evidence of the validity and reliability of the C-BiLLT in Dutch, Norwegian, and now Canadian children with CP, further research is needed to examine its validity and reliability among children with complex communication needs from different language and cultural backgrounds.

## Conflict of interest

JG developed the C-BiLLT and is currently head of the C-BiLLT foundation, which trains clinicians in Europe to use the tool. Profits are used to maintain the C-BiLLT’s software and online platform.

JWG, DM, DG, OC, BJC, and JG were awarded funds from the Hamilton Academic Health Sciences Network to support this work.

## Funding

This study was funded by the Hamilton Academic Health Sciences Organization Innovation Grant (HAH-18-003). Dr. Gorter held the Scotiabank Chair in Child Health Research at McMaster University during the execution of this study.
